# Jumping Height Does Not Increase in Well Trained Volleyball Players After Transcutaneous Spinal Direct Current Stimulation

**DOI:** 10.3389/fphys.2019.01479

**Published:** 2019-12-04

**Authors:** Łukasz Jadczak, Andrzej Wieczorek, Marcin Grześkowiak, Jacek Wieczorek, Dawid Łochyński

**Affiliations:** ^1^Department of Theory and Methodology of Team Sport Games, Poznań University of Physical Education, Poznań, Poland; ^2^Department of Pulmonological and Rheumatological Rehabilitation, Poznań University of Physical Education, Poznań, Poland; ^3^Department of Sport for Disabilities, Poznań University of Physical Education, Poznań, Poland; ^4^Department of Musculoskeletal Rehabilitation, Poznań University of Physical Education, Poznań, Poland

**Keywords:** countermovement jump height, squat jump, neuromodulation, spinal cord, jumping ability, spinal cord stimulation

## Abstract

Transcutaneous spinal direct current stimulation (tsDCS) increases corticospinal and spinal reflex excitability, and may be a new tool for increasing muscle explosive performance in sports training. The aim of the study was to evaluate whether tsDCS can enhance jumping ability in trained humans practicing volleyball. Twenty eight participants completed the study, including 21 men and 7 women. We investigated the effects of a single 15-minute session of sham, anodal, and cathodal tsDCS over spine and shoulder on repeated counter movement jump (CMJ) and squat jump (SJ) performance at 0, 30 and 60 min post-stimulation. The order of SJs and CMJs sets in each session was randomized. Each SJ and CMJ set consisted of 3 jumps. The break between each attempt was 1 min and the interval between the sets was 3 min. Two-way repeated measures ANOVA did not show effect of time, nor stimulation method, nor stimulation method × time interactions on SJ (time: *F*_(__1__.__8_,_142__.__1__)_ = 1.054; *p* = 0.346, stimulation: *F*_(__2_,_78__)_ = 0.019; *p* = 0.981, stimulation × time: *F*_(__3__.__6_,_142__.__1__)_ = 0.725; *p* = 0.564) or CMJ (time: *F*_(__1__.__8_,_140__.__9__)_ = 2.092; *p* = 0.132, stimulation: *F*_(__2_,_78__)_ = 0.005; *p* = 0.995, stimulation × time: *F*_(__3__.__6_,_140__.__9__)_ = 0.517; *p* = 0.705) performance. Single session of tsDCS over spine and shoulder does not increase jumping height in well-trained volleyball players. This is an important finding for coaches and strength conditioning professionals for understanding the practical utility of tsDCS for enhancing muscular explosiveness.

## Introduction

In volleyball, athletes and coaches pay special attention toward testing and developing maximum jumping ability to advance the level of playing (Sheppard et al., 2011). During the game the highest percentage of jumps is performed by the players in order to block, then to attack, and lastly to serve the ball (Vilamitjana et al., 2008). The higher the vertical jumping reach over the net during serving or spiking (e.g., attack jump) the better spiking or serving success (Sattler et al., 2015). Moreover, increase in vertical leap during blocking attempt decreases the effectiveness of attacks of the opposing team. During a volleyball game, players usually use two types of jumps without approach and arm swing to block a ball during the opponent attack. The first type is without (squatting jump, SJ) and the second is with a preliminary lower limb counter-movement (counter-movement jump, CMJ). The latter is a critical game element to obtain the optimal jumping range (Sheppard et al., 2008).

Squatting jump performance is dependent on the strength of neural activation which drives the muscles to action, and is primarily related to synchronic activation and recruitment intensity and motor units firing (Milner-Brown et al., 1973; Moritani and Muro, 1987). CMJ additionally involves a stretch–shortening cycle (SSC) resulting from the combination of eccentric and concentric muscle action (Komi, 1984). Apart from descending neural activation, vertical CMJ performance has been attributed to both elastic energy and reflex potentiation (Bosco et al., 1982). The peripheral Ia afferents from muscle spindles, the major contributors to the stretch reflex, have a net facilitatory influence on motoneurons increasing their discharge frequencies during motor tasks (Macefield et al., 1991). Therefore, high stretch reflex muscle activity is expected after a powerful stretch of the muscle–tendon complex (Avela and Komi, 1998).

Transcutaneous spinal direct current stimulation (tsDCS) is a non-invasive stimulation technique that can modulate activity of neurons within spinal cord (Priori et al., 2014). Anodal tsDCS was shown to increase spinal reflex excitability by inducing progressive leftward shift of the recruitment curve of spindle Ia afferent monosynaptic muscle reflex (Winkler et al., 2010; Lamy et al., 2012). On the other hand, increased corticospinal excitability and motor unit recruitment in the limb muscles was reported in healthy young adults after a single session of cathodal tsDCS (Bocci et al., 2014, 2015).

Very recently it has been reported that anodal transcranial DCS can enhance muscle power in men experienced with advanced strength training (Lattari et al., 2017). In the current study we aimed to verify if tsDCS increases maximum jumping height in volleyball trained humans. As cathodal tsDCS increases corticospinal drive to muscles while anodal tsDCS spinal reflex excitability, it was assumed that both would potentiate jumping performance through these specific neural mechanisms.

## Methods

### Experimental Approach

**FIGURE 1:**
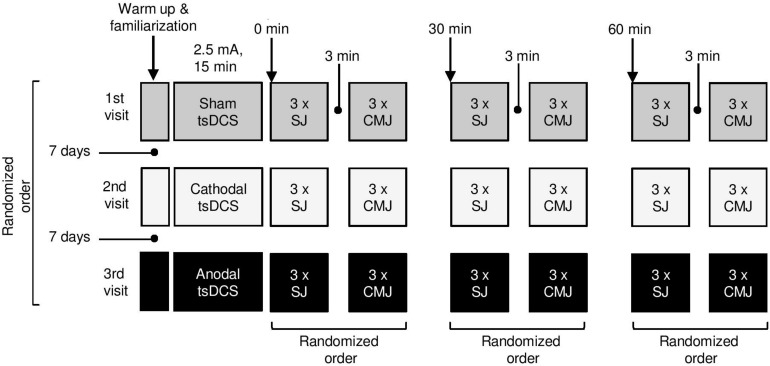
The experimental design.

This was a randomized crossover double-blind study in which each participant was subjected to a single sham, anodal, and cathodal tsDCS stimulation (independent variables) sessions in randomized order with 1 week apart. We tested the effects of local positive and negative spinal cord polarization on maximum SJ and CMJ height (dependent variables, Figure 1). This within subject study design allowed comparisons of jumping ability at the same periods after the anodal, cathodal, and sham stimulation which served as a reference measurement. We used such approach because it has been already demonstrated that anodal or cathodal stimulations were able to induce significant post tsDCS neural modulations while sham stimulation was not (Cogiamanian et al., 2008; Lamy et al., 2012). Both participants and researchers did not know which stimulation was administered at the time of taking the measurements and data analysis. This was achieved through coding the active and passive stimulation allocations for each condition and subject by an independent researcher prior to the start of the study.

### Participants

Thirty one subjects volunteered to participate in the present study. Three subjects were lost to follow up due to personal reasons or methodological errors. Ultimately, a total of 28 young healthy volleyball players (age: 22.1 ± 1.9 year old; weight: 75.0 ± 12.5 kg; height: 181.1 ± 7.2 cm; body mass index: 22.6 ± 2.6 kg × m^–2^; 21 male, age: 22.6 ± 1.8 year old; weight: 78.9 ± 11.5 kg; height: 184.0 ± 5.2 cm; body mass index: 23.2 ± 2.6 kg × m^–2^ and 7 females, age: 20.5 ± 1.4 year old; weight: 63.2 ± 6.6 kg; height: 172.2 ± 4.4 cm; body mass index: 21.3 ± 2.3 kg × m^–2^; means ± SD) completed the entire study protocol. Inclusion criteria for this study were as following: at least 7 years of experience in volleyball practice, and at least 2 h of involvement in training per day. Subjects with cardiovascular, respiratory and neuromuscular conditions or musculoskeletal injuries that could interfere on the study were excluded. We also excluded those with any implants inside the body (metal or electrical) to prevent harmful tsDCS interference. Subjects were instructed to abstain from caffeine at least 12 h before exercise. The examinations were performed on players who did not report any muscle damage resulting from preceding training loads. Research was carried out during the starting period of the volleyball season between the February and May 2017.

According to the International Physical Activity Questionnaire the participants were classified as vigorously active (Biernat, 2013). The protocol of the study was approved by the Bioethical Committee of Poznań University of Medical Sciences (decision letter no. 73/17), and was in accordance with the Declaration of Helsinki. All participants signed a written informed consent at the time of enrollment to the study.

### Training Program

The examined persons performed trainings every day from Monday to Friday from 4 to 6 pm, according to a weekly micro cycle characteristic for the so-called starting period in volleyball.

In the first day of each week the training content included stretching, relaxation and non-volleyball low-load exercises. In the next days, there were tactical volleyball exercises and also exercises to improve physical fitness with heavy loads. One training per week was devoted to develop jumping performance to increase players spiking and blocking ability. In the other days of the week training also involved exercises aimed at improvement of technique and tactics of the game (taking into account either own or the opponent’s game). Players played one and sometimes two matches per week. This training routine was the same throughout all weeks of the study.

### Procedure

#### Transcutaneous Spinal Direct Current Stimulation

**FIGURE 2:**
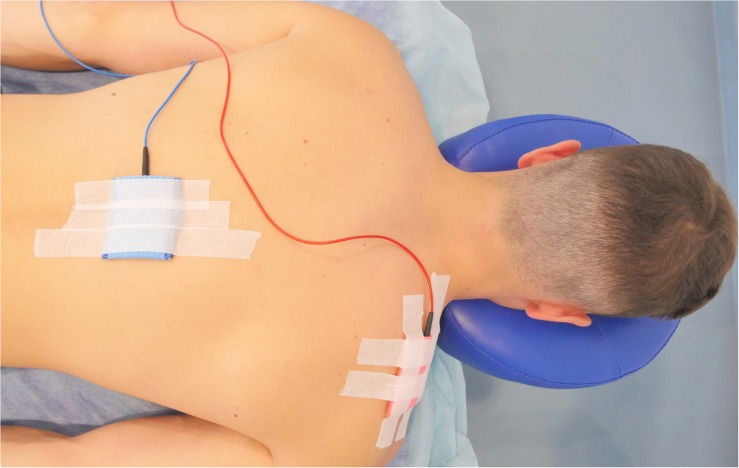
Schematic of the stimulation set-up.

Participants were asked to comfortably lay prone (Truini et al., 2011) on a portable massage table during the stimulation. TsDCS (2.5 mA, 15 min) was delivered by a pair of rectangular electrodes covered with saline-soaked sponges (7 × 5 cm, 35 cm^2^). One electrode was centered over the space between the 11th and 12th spinous processes of thoracic vertebrae (active anode or cathode) (Hubli et al., 2013), and the other (passive) above the right shoulder on the trapezius muscle (Bocci et al., 2015; Figure 2). The short axis of active electrode spanned the skin area from 11th to 12th thoracic vertebral bodies in order to cover longitudinally all five lumbar spinal cord segments (Sayenko et al., 2015). The positions of the electrodes were marked on the skin, and then transferred together with the skin distinguishing marks to the transparent foil. This was done to provide the electrode location record for subsequent treatments. Electrodes were connected to a constant-current programmable electrical stimulator (neuroConn, Ilmenau, Germany). During active stimulation over the spinal cord (anodal or cathodal) the current was ramped up to a target intensity over a 10 s period, than held constant over the stimulation period (900 s), and ramped down for 10 s at the end of session to minimize subject discomfort. The current density delivered during active stimulation was 0.071 mA/cm^2^ and provided a total charge of 63 mC/cm^2^. Based on previous recommendations such current was below the threshold for tissue damage (Nitsche et al., 2003). During the sham tsDCS, anode or cathode electrodes were placed in randomized order over the spinal cord to prevent the identification of stimulation by the researcher responsible for montage of electrodes. Similarly as for active stimulation the current was ramped up for 10 s, kept for 30 s and then ramped down for 10 s and maintained off during the rest of the stimulation period. Sham (placebo) stimulation was considered as a negative control condition in which an initial itching sensation was similar as during active stimulation but no current was flowed throughout the rest of the session. Except one subject who reacted with short lasting (several minutes) itching and skin redness under the site of electrode placement over the shoulder, other participants did not report any adverse events following the stimulation.

### Jumping Height Estimation

Jump height was quantified using OptoJump Next system equipment (Microgate, Bolzano, Italy). This system is composed of 100 × 4 × 3 cm bars with a transmitting and receiving sensors. Distance between the bars was 2 meters to accommodate the diversity of approaches. Each of the bars contains 96 leds (1.0416 cm resolution). Optojump bars were connected to a personal computer, and the proprietary software (Optojump software, version 1.12.1.0.) allowed jump height quantification. The Optojump system sampled the flight time with an accuracy of 1 kHz. Jump height was then estimated as 9.81 (gravity acceleration) × flight time^2^/8.

Prior to testing, all athletes completed a standardized 5-min warm up on a stationary bike (Monark 874E, Sweden) with pedaling rhythm of 50–60 revolutions per minute and without any load. Then, as a familiarization session, each subject performed two voluntary vertical SJs and CMJs according the audiovisual instructions describing the proper technique of the jumps. Before the first SJ experimental session subjects were instructed to perform a squat until the angle between the femur and leg was 90°. This position was adjusted using angle ruler. Its axis of rotation was aligned with the center of knee joint rotation at the lateral articular space. One arm of the ruler was directed to the greater trochanter of the femur and the other to the lateral malleolus of the leg. After setting the knee angle at 90°, a custom made height adjustable automatic seat was placed under the buttocks. Its vertical height was adjusted to enable only a light contact with ischial tuberosities. The determined seat height was stored for the subsequent experimental sessions to allow execution of SJ from the same starting position all over the study.

The CMJ was performed from an upright standing position, with self-chosen distance between both feet. After dynamic bending of the limbs to the half-squat position (counter movement preparatory phase) subject immediately performed hip, knee and ankle extension to elevate the body over the ground. Hands were kept on hips in both jumping conditions. The task was to perform the highest jump as possible.

Each jump was performed according to the following procedure: (1) on the command “enter”, the participant entered into the area between the Optojump bars and assumed the testing position; (2) on the command “go,” the subject performed the maximum jump; (3) the “exit” command completed the test. On each testing day, a participant performed 3 sessions composed of 3 sets of maximum SJs and CMJs, that is immediately after the tsDCS stimulation session and after 30 and 60 min of its completion. The order of SJs and CMJs sets in each session was randomized. In each set, the break between each jumping attempt was 60 s, and the interval between the SJ and CMJ sets was 3 min (Sattler et al., 2012). The jumps were carried out on the polyurethane-resin floor in the University sports research laboratory under the same conditions (22°C), at the same time of day. The entire experimental session lasted approximately 2 h. To ensure approximately similar recovery time before testing sessions the training in the preceding day was ended no later than 6 pm.

Visual analogue scale (VAS) was used to control the pain intensity related to the everyday volleyball training routine. This was done to exclude the potential influence of training related muscle soreness on jumping performance. Subjects were asked to mark on the VAS the current pain intensity of the lower limb musculature before each tsDCS stimulation.

### Statistical Analyses

The *a priori* sample size was estimated using G^∗^Power software (version 3.1.9.2; Kiel University, Kiel, Germany) (Faul et al., 2007). The statistical analysis was made in Statistica 13 (StatSoft Polska, Kraków, Poland). Repeatability analysis was performed on a subset of 252 samples obtained from each participant at three different time points and at three different tsDCS sessions. After having tested the existence of a normal distribution of the data sets, the interclass correlation coefficient (ICC) was used for the analysis. Mean estimations along with 95% confidence intervals (CIs) were reported for each ICC. For changes in jump height over time after three tsDCS stimulations a fixed effects ANOVA – special, main effects and interactions, with a “large” effect size *f* = 0.4, an α level = 0.05, power (1 – β err prob) = 0.8 and *df* = 4 [(3−1) × (3−1)] were assumed. The calculation estimated that at least 80 participants were necessary. However, due to limited resources of volleyball trained subjects, 31 participants were recruited to the study. The Shapiro–Wilk test was used to check the data normality, and Levene’s test to assess the equality of variances. The highest jump from the three performed was analyzed. For SJ and CMJ jumping vertical height a 3 × 3 ANOVA was performed with stimulation type (a-tsDCS; c-tsDCS and sham-tsDCS) and time (immediately, 30 and 60 min post stimulation) as factors. The sphericity assumption was tested using the Mauchly’s test and the Greenhouse–Geisser’s correction was used whenever data sphericity was violated. The comparison of VAS pain between the three tsDCS sessions was performed with the one-way analysis of variance. The level of significance was set at *p* < 0.05.

## Results

The ICC values of the recorded SJ and CMJ jumps were very high (range from 0.989 to 0.998), 95% CIs (lower range from 0.981 to 0.997, upper range from 0.995 to 0.999) for SJ and CMJ for each tsDCS condition and time post stimulation.

The pain of the lower limb musculature reported by the participants was very low and amounted to 1.0 ± 1.4, 0.8 ± 1.2 and 0.9 ± 1.1 points of the VAS before the sham, anodal and cathodal jumping sessions, respectively (*F*_(__2_,_84__)_ = 0.167; *p* = 0.846).

**FIGURE 3:**
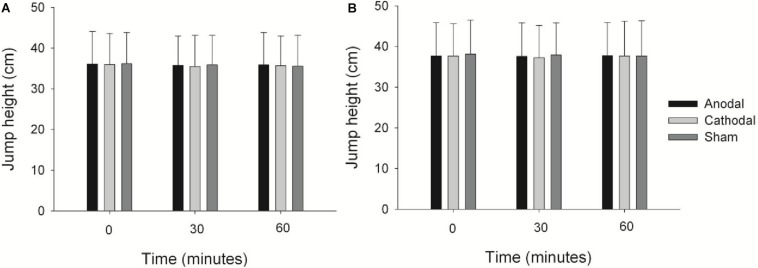
Jump height immediately after (0), 30 and 60 min post stimulations for SJ **(A)** and CMJ **(B)**. Values are means and standard deviations.

Neither sham, nor anodal, nor cathodal tsDCS did increase jumping height in well-trained volleyball players (Figure 3). The two-way ANOVA didn’t show effect of time, stimulation nor stimulation × time interactions for SJ and CMJ (Table 1).

**TABLE 1:** Summary ANOVA results on effects of stimulation on jump performance.

	**CMJ**				**SJ**			
		
	***F***	***df***	***p***	**η_p_^2^**	***F***	***df***	***p***	**η_p_^2^**
Stimulation	0.005	2, 78	0.995	0.000	0.019	2, 78	0.981	0.000
Time	2.092	1.8, 140.9	0.132	0.026	1.054	1.8, 142.1	0.346	0.018
Stimulation x time	0.517	3.6, 140.9	0.705	0.013	0.725	3.6, 142.1	0.564	0.013

*CMJ, counter movement jump; SJ, squat jump; df, degrees of freedom; (η_*p*_^2^), partial eta squared (effect size).*

## Discussion

In this study tsDCS was applied to verify, if in the future it can constitute an additive method to strength or plyometric training (Voelzke et al., 2012) to develop explosive muscle force and enhance jumping performance in volleyball players (Puhl et al., 1982). However, neither cathodal nor anodal single session of tsDCS enhanced SJ or CMJ performance in volleyball trained individuals.

The intensity of pain in lower limb musculature was assessed before each tsDCS session. This was done to exclude the possibility that jumping height could be affected by the delayed muscle soreness resulting from regular training activities, and to ensure that the potential training load variations preceding each testing day does not affect the effects of tsDCS on jumping performance. VAS scores showed that the experienced pain was negligible and similar before each testing day. This indicates, that pain has not affected the jumping height in the studied players.

Acute explosive muscle performance can be potentially enhanced by an increase in recruitment of especially high-threshold units, which contain fast-contracting muscle fibers (Sale, 1988), or by the increase in synchronization of discharge activity of concurrently active motor units (Milner-Brown et al., 1975). Jumping performance can be also improved by increase in maximum firing rates of recruited motor units or increased incidence of brief, high frequency bursts of motor unit action potentials (Desmedt and Godaux, 1977) during explosive muscle contractions. These modifications in neural activity have been previously considered as potential mechanisms allowing quicker attaining of the peak muscular force or increasing the force produced by motor units (Sale, 1988). Based on our data it can be indirectly supposed that neither cathodal tsDCS, which has been reported to increase corticospinal excitability and motor unit recruitment (Bocci et al., 2014, 2015), nor anodal tsDCS, which has been reported to increase spinal reflex excitability, were able to induce these specific neural modifications to increase jumping performance in volleyball trained humans. One working hypothesis is that in explosively trained athletes, jumping performance cannot be further enhanced by functional neural modifications. For instance, the maximum limits of motor unit recruitment could be already reached in trained volleyball players, because during explosive movements, the activation threshold of motor units is decreased, as their recruitment thresholds decrease progressively with increase in the speed of muscle contraction (Desmedt and Godaux, 1977). Moreover, dynamic training induces adaptive enhancement of maximal firing rates of motor units and increases the incidence of double discharges during ballistic contractions (Van Cutsem et al., 1988). Furthermore, dynamic training seems to change electrophysiological properties of motoneurons toward the faster profile (Casabona et al., 1990). Hence, it might be that tsDCS is unable to further enhance neuromuscular activity in well trained volleyball players.

In our study we applied the most common tsDCS montage, which allowed spine to shoulder current flow and has been shown to induce various neuromodulatory effects in previous human studies (Priori et al., 2014). Very recently a single session of anodal tsDCS has been shown to enhance countermovement vertical jumping performance with arm swing in healthy non-athletes (Berry et al., 2017). In that study, transabdominal tsDCS was applied as, based on computer modeling, it was identified that this method is capable to deliver a higher density and more focused current over the lumbosacral cord when compared to the spine-shoulder tsDCS (Parazzini et al., 2014). In theory, the change in excitability of neurons may depend on the orientation of the neuronal compartments located in close proximity to the active electrode (Ahmed, 2011). Therefore, an alternative hypothesis behind the lack of changes in jumping performance is that the applied layout of electrodes was not optimal to induce the desired neuromodulatory effects in the current study.

The major limitation of the current study is that, due to limited resources, we were unable to collect the data from the total number of participants, who were *a priori* estimated to constitute an appropriate sample size. This leaves some possibility, that type II statistical error might occur and lead to the false negative findings. However, the jumping height was very consistent between the different stimulations. Therefore, we suppose that even if a small difference was observed after the sample size was enlarged, this would not necessarily have any practical significance for the jumping performance.

We have expected that spinal cord stimulation can be a novel, safe and legal agent which will stimulate jumping to exceed gains resulting from physical exercises incorporated in regular volleyball training. From the practical perspective, a minor increase in maximum jump height just after a single stimulation session would be a relevant achievement. For example we believe that increase in players’ physicality during a single volleyball match would be of great value for achieving better results in volleyball competition. Although no positive effects were found after a single session, it seems premature to claim that tsDCS is ineffective in enhancing jump height. There are many modifications in DCS administration, which can increase its effectiveness. In future, the arrangement of electrodes on the body should be manipulated to determine the most optimal location for enhancing spinal cord excitability. Also, it would be beneficial to know if longer session of tsDCS results in higher jumping height. Furthermore, other more targeted and focal stimulation techniques, such as these which turned out to be effective in increasing human cortical excitability (Elsner et al., 2018), could be adapted to modulate spinal cord circuitry and enhance muscle explosive capabilities. Finally, it would be also interesting to verify if repeated stimulation combined with the regular physical training is able to induce neural plasticity, which results in greater muscle explosiveness.

In conclusion, the present study shows that jumping height is not enhanced after spine-shoulder tsDCS in well trained volleyball players. This is an important finding for understanding the practical utility of tsDCS in sports training. These results undermine the rationale for use of tsDCS in volleyball training practice. Nevertheless, it remains to be elucidated if tsDCS is not capable to enhance jumping ability or the negative findings are caused by specific application of tsDCS, which does not target appropriate neural compartments and pathways.

## Data Availability Statement

The datasets generated for this study are available on request to the corresponding author.

## Ethics Statement

The studies involving human participants were reviewed and approved by the Bioethics Committee of Poznań University of Medical Sciences. The patients/participants provided their written informed consent to participate in this study.

## Author Contributions

DŁ, ŁJ, and AW designed the experiments. All authors collected, analyzed, and interpreted the data.

## Conflict of Interest

The authors declare that the research was conducted in the absence of any commercial or financial relationships that could be construed as a potential conflict of interest.
